# The effect of co-morbidities on health-related quality of life in patients placed on the waiting list for total joint replacement

**DOI:** 10.1186/1477-7525-5-16

**Published:** 2007-03-15

**Authors:** Ulla Tuominen, Marja Blom, Johanna Hirvonen, Seppo Seitsalo, Matti Lehto, Pekka Paavolainen, Kalevi Hietanieni, Pekka Rissanen, Harri Sintonen

**Affiliations:** 1 National Research and Development Centre for Welfare and Health, Helsinki, Finland; 2 University of Helsinki, Finland; 3 HUCH Hospital Area, Espoo, Finland; 4 Academy of Finland; 5 Orton Orthopaedic Hospital, Helsinki, Finland; 6 Coxa, Hospital for Joint Replacement, Medical Research Fund of Tampere University Hospital, Tampere, Finland; 7 University of Tampere, Finland

## Abstract

**Background:**

Co-morbidity is a powerful predictor of health care outcomes and costs, as well as an important cofounder in epidemiologic studies. The effect of co-morbidities is generally related to mortality or complications. This study evaluated the association between co-morbidity and health-related quality of life (HRQoL) in patients awaiting total joint replacement.

**Methods:**

A total of 893 patients were recruited to the study between August 2002 and November 2003 in four Finnish hospitals. The effect of co-morbidity on HRQoL was measured by the generic 15D instrument and by a Visual Analog Scale (VAS). Comparative variance analysis of socio-demographic and clinical characteristics was described by using either an independent samples t-test or the Chi-square test. The differences in each of the 15D dimensions and the overall 15D single index score for patients were calculated. Two-sided p-values were calculated with the Levene Test for Equality of Variances.

**Results:**

Patients with co-morbidity totaled 649; the incidence of co-morbidity was 73%. The mean number of co-morbidities among the patients was two. At baseline the 15D score in patients with and without co-morbidity was 0.778 vs 0.816, respectively. The difference of the score (0.038) was clinically and statistically significant (P < 0.001). The patients' scores with and without co-morbidity on the different 15D dimensions related to osteoarthritis-moving, sleeping, usual activities, discomfort and symptoms, vitality and sexual activity–were low in both groups. Patients with co-morbidity scored lower on the dimensions of moving, vitality and sexual activity compared to the patients without co-morbidity. Co-morbidity was significantly associated with a reduced HRQoL. Patients without co-morbidity had poorer VAS, arthritis had strong effect to their quality of life compared to the patients with co-morbidity.

**Conclusion:**

Assessing co-morbidity in patients placed on the waiting list for joint replacement may be useful method to prioritization in medical decision-making for healthcare delivery. The assessment of co-morbidities during waiting time is important as well as evaluating how the co-morbidity may affect the final outcomes of the total joint replacement.

## Background

Chronic diseases have been shown to negatively affect people's quality of life (QoL), and they are a common reason for disability and early death. According to the Finnish National Health 2000 Survey half of the Finnish population aged over 30 has at least one chronic disease, while 44 % of the working population and 82 % of senior citizens have at least one chronic disease [1]. The decline of the populations' self-reported sense of well-being is a consequence of ageing, with no observable difference between women and men. The commonest chronic diseases in Finland are: cardiovascular disease, musculoskeletal disease, diabetes, and lung diseases [1].

Co-morbid or coexisting disease refers to the occurrence of two or more diseases in the same individual. The presence of co-morbidity has a pervasive effect on QoL, research, and clinical practice through its influence on diagnosis, prognosis, treatment and, health care delivery. Each co-morbid disease may have its own effect on QoL while also having a clinical effect on patients' sense of well-being. It is also important for studies of patients with chronic disease in whom mortality is rare and the goal of medical care is to control the course of the disease and maximize the quality of life [2]. Evaluations on how different diseases affect patients' health-related quality of life (HRQoL) focus mostly on the index disease, considering the effect of co-morbidities to a lesser extent. However when the focus is on the consequences–mortality, complications or in the costs of the medical care – co-morbidity becomes an important denominator.

Co-morbidity can also play an important role in different types of studies. Randomized controlled trials and prognostic studies might be complicated by co-morbidity. It can either act as a cofounder, threatening the internal validity, or as an effect modifier, threatening the internal and external validity of the study: therefore an efficient method is needed to measure co-morbidity [3].

In Finland, total joint replacements (TJR) are surgical procedures with high volume and long waiting times. In 2003, primary hip and knee replacements were carried out for almost 15500 patients [4]. For patients with primary total joint replacement of hip the median waiting time was 155 days, and for patients with primary total joint replacement of knee, 205 days [4]. One reason for these long waiting times is that OA is not itself life threatening. However, some previous studies have reported that those awaiting hip or knee replacement have a significantly poorer quality of life and that arthritis becomes a chronic and heavy burden to the patients [5,6]. Also few studies having examined waiting time effects on health status in OA patients have not been able to show that patients having to wait longer would suffer from pain and functional difficulties or poorer HRQoL than those with shorter waiting [7,8].

In 2002, a prospective multi-centre study was started in four Finnish hospitals. The aim of this larger study is to assess the effect, the costs and the cost effectiveness of the waiting time in patients awaiting TJR. This report is part of this ongoing study. The objective of this paper is to evaluate the effect of co-morbidity on HRQoL in OA patients when placed on the waiting list for TJR.

## Methods

### Data collection

Between August 2002 and November 2003, a total of 893 OA patients were enrolled in the study in four Finnish hospitals: the Helsinki University Central Hospital: Surgical Hospital, the Helsinki University Central Hospital: Jorvi Hospital, the Coxa Hospital for Joint Replacement and the Orton Orthopedic Hospital. Patients were recruited into the study through contact with orthopedic and practice staff.

The key inclusion criteria were a need for a primary TJR due to OA of the hip or knee joint as evaluated by the hospital surgeon; patient was aged 16 or older and placed on the waiting list in a research hospital, and the patient was willing and mentally able to participate in the study. The key exclusion criteria were patients with rheumatoid arthritis, congenital hemophilia or congenital deformities, and fractures. Patients completed a self-administered questionnaire when placed on the waiting list for TJR. The questionnaires were distributed to the patients at the hospital and were returned by post. Common guidelines for administering the questionnaires were provided at each hospital. The patients completed a socio-demographic form, reported their co-morbidities as diagnosed by a medical doctor, completed the visual analog scale (VAS), and also completed separate questionnaires for self-reported sense of well-being and HRQoL. Each patient provided informed consent. The study was approved by the Helsinki University Central Hospital Surgery Ethics Committee.

In this study, the co-morbidity data was collected from the patients' reported co-morbidities as diagnosed by a medical doctor. The patients were assigned to subgroups according to co-morbidity status. The patients' reported diseases were classified according to the ICD10 [9], giving nine diagnoses groups in total: tumors, diabetes mellitus, respiratory disease, cardiovascular disease, high cholesterol, mental health problems, muskuloskeletal system diseases, endocrinological problems, and visual or hearing problems.

### Co-morbidity and Quality of Life

It is difficult to choose the most appropriate co-morbidity measurement because comparative data on how the available instruments perform in different disease settings are limited [10,11]. There are several reports about different measurements and measurement combinations for assessing co-morbidity. Instruments used in clinical research to calculate co-morbidity include, for example, the Carlson Index [13], the Cumulative Illness Rating Scale (CIRS) [14], the Index of Coexistent Diseases (ICED) [15] and the Kaplan Index [16]. Both generic and disease-specific QoL instruments are used to assess the effect of co-morbidities in clinical trials [12,17].

In this study the effect of co-morbidity was assessed as the difference in HRQoL between the patients with and those without co-morbidity. HRQoL was measured by using the generic, multidimensional, standardized, and self-administered 15D instrument. The 15D is a Multi-Attribute-Utility-Scale (MAU) measurement instrument [18] that measures quality of life in 15 dimensions: moving, vision, hearing, breathing, sleeping, eating, speech, eliminating, vitality, mental functions, discomfort and symptoms, depression, distress, energy and sexual activity. Each dimension has a single question with 5 possible answer options. The 15D can be used as a profile measure or to give a single index score by means of population-based preference weights. The index score is between 0 (being dead) and 1 (being totally healthy). Completing the 15D questionnaire takes 5–10 minutes and it describes the respondents HRQoL at that point in time. The minimum clinically important difference (MCID) in the 15D single index score is interpreted as a difference of ± 0.03 or more, which corresponds to the minimum difference that people can generally distinguish [19]. The 15D favourably compares with other quality of life instruments–such as EuroQol (EQ-5D), Health Utility Index (HUI 1–3), Short Form- 36 (SF-36), and the Nottingham Health Profile (NHP)–in most of the important properties (e.g. responsiveness, reliability and validity) [20-22].

To evaluate the patients' sense of well-being, we used VAS, which is a health-state rating scale. The measurement consists of a line on a page with clearly defined endpoints. The most preferred health state is placed at one end of the line and the least preferred at the other end [23,24]. In this study, VAS was a horizontal 100 mm long line (100 mm= 100%) illustrating the patients' health state deficiency at that moment. It was used to evaluate the effect of arthritis on health. Patients were asked to mark on the line which part of the deficiency of health is due to arthritis. The higher the number was–on scale from 0 to 100–the more powerful was the effect of arthritis. In addition, the patients' self-reported state of health was described with a five-point scale, representing health states from excellent to worst.

### Statistical analysis

At the baseline (when placed on the waiting list) descriptive statistics were used to describe the socio-demographic and clinical characteristics of the patients. Comparative variance analyses of socio-demographic and clinical characteristics were described by using either an independent samples t-test or the Chi-square test depending on whether it was a continuous or nominal scale. The differences in each of the 15D dimensions and the overall 15D single index score for patients were calculated. Two-sided p-values were calculated with the Levene Test for Equality of Variances, with the minimum significance level set at 5% (P-value < 0.05). The mean differences between each of the dimensions were also calculated. Missing values for the 15D were predicted by means of a regression model with the patient's responses for other dimensions, and also with data from the patients, with age and gender as explanatory variables [22]. The missing values were estimated if a minimum of 80% of dimensions had been completed. Furthermore, the incidences of co-morbid diagnoses and also the mean 15D score were calculated for each diagnosis group in the model. Data analyses were performed using SPSS version 12.0.1 for Windows.

## Results

Of the 914 eligible patients recruited into this study, twenty one were excluded because they didn't return the questionnaire, leaving 893 patients in the study group. The mean age of patients waiting for TJR was 66 years (range 24–88) and 63% of the participants were female. Patients with co-morbidity totaled 649, while patients without co-morbidity totaled 244. The mean age in the patients with co-morbidity was 67 years (range 25–87), versus 64 years (range 24–88) without co-morbidity (P < 0.001). Patient's demographics are reported in Table 1. The incidence of co-morbidity as a secondary or tertiary illness was 73%. The mean number of co-morbidities among the patients was two, and 363 (56% of co-morbid patients) had three or more diagnosis. The BMI was high in both groups (>25 which is the limit of overweight and >30 is a limit of obese) but in the co-morbid group, the BMI was higher than in the patients without co-morbidity (P < 0.001). The patients' health state based on the five-point scale was worse in the co-morbidity group. However in the patients with co-morbidity, the effect of the OA in the health-state deficiency as measured by VAS was 62% versus 76% (P < 0.001) in the patients without co-morbidity (Table 1).

**Table 1:** Demographic and clinical characteristics of TJR patients with and without co-morbidity when placed on the waiting list. Independent Sample T-test or Chi-square test

**Characteristics**	**Patients with co-morbidity**	**Patients without co-morbidity**	**P-value**
	(n = 649–662^1^)	(n = 242–244^1^)	
Age, years (mean ± SD)	67 ± 10	64 ± 11	0.000***
Females [n, (%)]	422(63)	132 (54)	0.008**
Marital status [n, (%)]			
Married	411 (62)	160 (65)	0.155
Housing [n,%]			
Living alone	227 (34)	62 (25)	0.008**
Basic education [n,%]			
Low level	544 (82)	190(78)	0.130
Professional examination [n,%]			
Low level	486 (75)	175 (73)	0.279
Employment status {n, (%)]			
Retired	546 (82)	172(71)	0.000***
Health status [n, (%)]			
Fair or poor	497 (75)	133 (55)	0.000***
VAS^2 ^(mean ± SD)	62 ± 26	76 ± 24	0.000***
BMI^3 ^(mean ± SD)	28.8 ± 4	27.2 ± 4	0.000***

^1^Number of observation varies due to missing values; ^2^VAS, visual analog scale (100 the worst, 0 the best value); ^3^BMI, body mass index (wt/ht^2^); ** P < 0.01, ***P < 0.001

The most common secondary diagnosis was cardiovascular disease (n = 419, 63%), followed by high cholesterol (n = 225, 33%), diabetes mellitus (n = 225, 33%) and endocrinological problems (n = 225, 33%). The worst 15D score of 0.637 was for a patient with mental problems (Table 2).

**Table 2:** The co-morbidities among osteoarthritis patients and the mean 15D score in each diagnose groups, when placed on the waiting list for TJR

Co-morbidity	Patients, n (%)	15D (± SD)
Cardiovascular disease	419 (63%)	0.775 (± 0.09)
High cholesterol	225 (34%)	0.769 (± 0.09)
Diabetes mellitus	225 (34%)	0.769 (± 0.09)
Endocrinological problems	225 (34%)	0.769 (± 0.09)
Other muskuloskeletal diseases	117 (18%)	0.758 (± 0.09)
Respiratory diseases	91 (14%)	0.758 (± 0.09)
Visual or hearing problems	54 (8%)	0.780 (± 0.07)
Tumors	25 (4%)	0.735 (± 0.08)
Mental problems	14 (2%)	0,637 (± 0.09)

At baseline the 15D score in patients with and without co-morbidity was 0.778 vs 0.816, respectively. The difference of the score (0.038) was clinically and statistically significant (P < 0.001). The patients' scores with and without co-morbidity on the different 15D dimensions related to OA–moving, sleeping, usual activities, discomfort and symptoms, vitality and sexual activity–were low in both groups. Patients with co-morbidity scored lower on the dimensions of moving, vitality and sexual activity compared to the patients without co-morbidity, while patients without co-morbidity scored lower in the dimensions of seeing, hearing, breathing and elimination. The deterioration of HRQoL was significantly associated with co-morbidity (Table 3).

**Table 3:** 15D dimensions and score at baseline in total joint replacement patients withand without co-morbidity. Levene's Test for Equality of Variances

**Dimensions**	**Patients co-morbidity (n = 662)**		**Patients no co-morbidity (n = 242)**			
	Mean	SE	Mean	SE	Mean difference	P-value

Moving	0.590	0.137	0.626	0.137	0.036	0.000***
Seeing	0.905	0.181	0.934	0.152	0.030	0.015*
Hearing	0.915	0.154	0.945	0.129	0.031	0.003**
Breathing	0.812	0.235	0.937	0.138	0.125	0.000***
Sleeping	0.723	0.217	0.738	0.210	0.014	0.375
Eating	0.991	0.057	0.997	0.032	0.006	0.051
Communication	0.986	0.070	0.993	0.046	0.007	0.102
Elimination	0.827	0.216	0.910	0.162	0.083	0.000***
Usual activities	0.643	0.222	0.679	0.239	0.036	0.045*
Mental	0.889	0.176	0.906	0.162	0.017	0.180
Discomfort and symptoms	0.506	0.236	0.540	0.240	0.033	0.065
Depression	0.837	0.171	0.854	0.167	0.016	0.196
Distres	0.848	0.177	0.865	0.162	0.017	0.168
Vitality	0.737	0.179	0.789	0.153	0.052	0.000***
Sexual activity	0.732	0.281	0.783	0.243	0.051	0.008**
15D-index	0.778	0.092	0.816	0.281	0.038	0.000***

*P < 0.05; **P < 0.01;***P < 0.001

## Discussion

The aim of this study was to assess the effect of co-morbidities on HRQoL at baseline in patients awaiting major joint replacement in four Finnish hospitals. The main finding of this study was that the HRQoL of all TJR patients was poor but significantly worse in the patients with co-morbidity. A secondary finding was that the VAS health-rating instrument appears more appropriate as a disease-specific instrument, as in this study, patients did not necessarily assign the primary disease (arthritis in most cases) as being the most prominent in affecting their well-being. Furthermore, the five-point self-rated health scale showed that the health state of patients with co-morbidity was worse than the patients without co-morbidity, which is in line with our results of a decline from the baseline of HRQoL as measured by 15D.

Few studies have assessed the effect of co-morbidities on the quality of life (QoL) by either a generic or a disease-specific instrument. In 1999 Xuan et al. [2] reported how different measuring methods differ when evaluating the different effects of co-morbidities related to QoL. They found that co-morbidity extensively affects generic QoL, whereas the effect is considerably smaller for disease-specific measures. Salaffi et al. [17] studied the relationship between OA, co-morbidity and HRQoL in older adults compared with matched healthy controls. They found that 55% of patients reporting at least one chronic coexisting disease and OA of the lower extremities suffer a significant impact on multiple dimensions of HRQoL compared with healthy controls. The most significant impacts were seen in physical functions, physical roles, and pain. Both of these findings are in line with our results.

Cardiovascular disease is the most common disease in Finland [1] and in our study, it was also the most common coexisting disease. According to Shan et al. [14] if OA is related in some way to a co-morbidity i.e. cardiovascular disease, it might negatively affect the outcomes of the joint replacement. Furthermore, several studies have testified that arthritis is considered to be a risk factor for other co-morbidity conditions such as hypertension, heart disease, diabetes and chronic lung disease [17,25].

Limitations of this study include the severity of the co-morbidity not being known, and the fact that arthritis was necessarily classified as the primary disease. However, we can assume that the patients' coexisting diseases were not life threatening, because the severity of the co-morbidity in the patients is usually an exclusion criterion for the surgical operation. The other limitation related to co-morbidity data was that the data was collected from the patients, not from the patient's medical records. However the patients were asked to name only those co-morbidities diagnosed by a medical doctor.

Co-morbidity has been commonly measured as an index in studies where medical records have been used to investigate mortality, complications or the costs of the medical care. A significantly strong association between co-morbidity and mortality, complications and increasing in hospital costs [2,12,14,25-28] has been shown, but to our knowledge there are no previous studies on the effect of co-morbidity on the HRQoL in the patients waiting for TJR.

Our findings show statistically significant differences in the 15D-index between the groups with and without co-morbidity. This suggests that a generic measurement instrument is sensitive enough to identify the effects of co-morbidities. This study provides evidence of co-morbidity being a factor that significantly affects HRQoL, and which can be assessed when the patients are placed on the waiting list. Moreover, some studies have suggested that the use of a generic HRQoL measurement in the studies of OA where co-morbidity is common would be useful in characterizing the global burden of this disease [16,17].

## Conclusion

Severity of OA was the only inclusion criteria of patients when placed on the waiting list, and the surgery was performed according to the hospital's routine procedure, this study had no effect to this. In these analyses, we found that the HRQoL of all TJR patients was poor but significantly worse in the patients with co-morbidity. Further, VAS health-rating instrument appears more appropriate as a disease-specific instrument, as being the most prominent in affecting patients' well-being, and also the five-point self-rated health scale showed that the health state of patients with co-morbidity was worse than the patients without co-morbidity. Assessing the co-morbidity condition at baseline might operate as an instrument to help in prioritization in medical decision-making for healthcare delivery. The assessment of co-morbidities during waiting time is important as well as evaluating how the co-morbidity may affect the final outcomes of the whole procedure.

## Competing interests

The author(s) declare that they have no competing interests.

## Authors' contributions

UT was the correspondence author of the manuscript and responsible for the integrity of the work as a whole. She contributed as a principal researcher and writer including drafting the article and the analysis and interpretation of the data. MB was the leader of the research project. She made contributions and design, acquisition and interpretation of the data and participated in the writing process by commenting the manuscript. JH made contributions to design, acquisition, and interpretation of data. HS and PR contributed as specialists in the field, and were involved in the design of the study and hypothesis formation. PP, SS, ML and KH contributed as specialists in the field of orthopaedic surgery. They made contributions to design and acquisition of data.

## References

[B1] NationalHealth Institution (2003). Health in 2000. Finish National Survey.

[B2] Xuan J, Kirchdoerfer LJ, Boyer JG, Norwood GJ (1999). Effects of comorbidity on health related quality of life. Scores: An analysis of clinical trial data. Clin Ther.

[B3] de Groot V, Beckerman H, Lankhorst GJ, Bouter LM (2003). How to measure comorbidity, a critical review of available methods. J Clin Epidemiol.

[B4] National Research and Development Centre for Welfare and Health Operative inpatients service 2003. http://www.stakes.info/2/9/hoitojaksot_2003.asp.

[B5] Hirvonen J, Blom M, Tuominen U, Seitsalo S, Lehto M, Paavolainen P, Hietaniemi K, Rissanen P, Sintonen H (2006). Health -related quality of life in patients waiting for major joint replacement. A comparison between patients and populations. Health Qual Life Outcomes.

[B6] Croft P, Lewis M, WynnJones C, Coggon D, Cooper C (2002). Health status in patients awaiting hip replacement for osteoarthritis. Rheumatology.

[B7] Kelly KD, Voaklander DC, Johnston DW, Newman SC, Suarez-Almazor ME (2001). Change in pain and function while waiting for major joint arthroplasty. J Arthroplasty.

[B8] Derret S, Paul C, Morris J (1999). Waiting for elective surgery: effect on health-related quality of life. Int J Qual Health Care.

[B9] International statistical Classification of Diseases and Related Health Problems (1994). ICD10. World Health Organization (WHO)I.

[B10] Imamura K, Black N (1998). Does comorbidity affect the outcome of surgery? Total hip replacement in UK and Japan. Int J Qual Health Care.

[B11] Gabriel S, Crowson C, O'Fallon (1999). A Comparison of two comorbidity instruments in arthritis. J Clin Epidemiol.

[B12] Greenfield S, Apolone G, McNeil BJ, Cleary PD (1993). The importance of co-existent disease in the occurrence of postoperative complications and one-year recovery in patients undergoing total hip replacement. Comorbidity and outcomes after hip replacement. Med Care.

[B13] Charlson ME, Pompei P, Ales KL, MacKenzie CR (1987). A new method of classifying prognostic comorbidity in longitudinal studies: Development and validation. J Chronic Dis.

[B14] Shah AN, Vail TP, Taylor D, Pietrobon R (2004). Comorbid illness affects hospital costs related to hip arthroplasty. Quantification of health status and implications for fair reimbursement and surgeon comparisons. J Arthroplasty.

[B15] Linn B.S, Linn M.W, Gurel L (1968). Cumulative illness rating scale. J Am Geriatr Soc.

[B16] Piccirillo JF, Louis St, MO (2000). Comorbidity as a New Data Element. Presentation to the Committee on Standards Commission on Cancer.

[B17] Salaffi F, Carotti M, Stancati A, Grassi W (2005). Health- related quality of life in older adults with symptomatic hip and knee osteoarthritis: comparison with matched healthy controls. Aging Clin Exp Res.

[B18] Brazier J, Derveril MI (1998). The use of health-related quality of life measures in economic evaluation Health Services Research Methods Guide to best practice.

[B19] Sintonen H (2001). The 15D measure of health-related quality of life: properties and applications. Ann Med.

[B20] Bowling A (2005). Measuring Health A review of quality of life measurement scores.

[B21] Sintonen H (1994). The 15d measure of health- related quality of life: reliability, validity and sensitivity of its health state descriptive system Working paper 41 NCHPE.

[B22] Sintonen H http://www.15d-instrument.net/15d.

[B23] McDowell P, Newell C (1996). Measuring Health A guide to rating scales and questionnaires.

[B24] Rissanen P, Aro S, Sintonen H, Slatis P, Paavolainen P (1996). Quality of life and functional ability in hip and knee replacements: A prospective study. Qual Life Res.

[B25] Mont MA, Mears SC, Jones LC, Rajadhyaksha AD, Krackow AM, Bawa M, Hungerford DS (2002). Is coding of diagnosis, comorbidities and complications in Total Knee Arthroplasty Accurate?. J Arthroplasty.

[B26] Bleichrodt H, Crainich D, Eeckhoudt L (2003). The effect of comorbidities on treatment decisions. J Health Econ.

[B27] Roche JJ, Wenn RT, Sahota O, Moran CG (2005). Effect of comorbidities and postoperative complications on mortality after hip fracture in elderly people: prospective observational cohort study. BMJ.

[B28] Paavolainen P, Pukkala E, Pulkkinen P, Visuri T (2002). Causes of death after total hip Arthroplasty. J Arthroplasty.

